# Temporal Requirements of cMyc Protein for Reprogramming Mouse Fibroblasts

**DOI:** 10.1155/2012/541014

**Published:** 2012-04-26

**Authors:** Corey Heffernan, Huseyin Sumer, Luis F. Malaver-Ortega, Paul J. Verma

**Affiliations:** ^1^Cell Reprogramming and Stem Cells Laboratory, Centre for Reproduction and Development, Monash Institute of Medical Research, Monash University, 27-31 Wright Street, Clayton, VIC 3168, Australia; ^2^South Australian Research and Development Institute, Turretfield Research Centre, Rosedale, SA 5350, Australia

## Abstract

Exogenous expression of Oct4, Sox2, Klf4, and cMyc forces mammalian somatic cells to adopt molecular and phenotypic characteristics of embryonic stem cells, commencing with the required suppression of lineage-associated genes (e.g., *Thy1* in mouse). Although omitting cMyc from the reprogramming cocktail minimizes risks of uncontrolled proliferation, its exclusion results in fold reductions in reprogramming efficiency. Thus, the feasibility of substituting cMyc transgene with (non-integrative) recombinant “pTAT-mcMyc” protein delivery was assessed, without compromising reprogramming efficiency or the pluripotent phenotype. Purification and delivery of semisoluble/particulate pTAT-mcMyc maintained Oct4-GFP^+^ colony formation (i.e., reprogramming efficiency) whilst supporting pluripotency by various criteria. Differential repression of Thy1 by pTAT-mcMyc ± Oct4, Sox2, and Klf4 (OSK) suggested differential (and non-additive) mechanisms of repression. Extending these findings, attempts to enhance reprogramming efficiency through a staggered approach (prerepression of Thy1) failed to improve reprogramming efficiency. We consider protein delivery a useful tool to decipher temporal/molecular events characterizing somatic cell reprogramming.

## 1. Introduction

Forced expression of four key transcriptional regulators, Oct4, Sox2, Klf4, and cMyc, converts mammalian somatic cells to “induced pluripotent stem cells” (iPSCs), that satisfy all pluripotent criteria of embryonic stem (ES) cells [1–3]. The reprogramming of fibroblasts occurs sequentially, commencing with requisite suppression of lineage-associated genes [4, 5]; **Thy1 ** (CD90) is a glycosylphosphatidylinositol-anchored plasma membrane glycoprotein expressed in murine fibroblasts and commonly used as a lineage gene marker in reprogramming literature [5–7]. Although required for endogenous lineage gene suppression [8], exogenous cMyc expression is dispensable for the induction of pluripotency, and its omission from the reprogramming cocktail favourable given its link to oncogenesis. However, fold-reductions in reprogramming efficiency commonly result (potentially due to maintenance of endoderm gene regulators and failure to activate microRNA clusters beneficial to reprogramming; [9–11]). Thus, application of nonintegrative cMyc conceptually circumvents risks of oncogenesis whilst utilising beneficial effects in regards to lineage gene suppression.

Application of fusion protein incorporating (i) a cationic polyarginine tag (for transduction across plasma membranes) and (ii) cMyc sequence, in combination with the other reprogramming proteins, to target cells *in vitro* successfully reprograms murine and human cells to pluripotency [12, 13]. These studies purified denatured protein from bacterial inclusion bodies before refolding and application to target cells (micromolar concentrations) [12], or applied unknown concentrations of whole protein extract from induced human cells without purification [13]. Initial attempts to purify recombinant proteins incorporating (i) reprogramming factor domains fused to (ii) a similarly arginine-rich basic domain (^49^ **R**KK**RR**Q**RRR** ^57^) of HIV transactivating transcriptional-activator (Tat) protein from bacterial inclusion bodies under denaturing conditions encountered problems with restriction to endosomes in target cells following transduction [14]. Binding of the Tat transduction domain to plasma membrane-bound heparan sulfate proteoglycans initiates transduction through caveolar (“lipid raft”) endocytosis; translocation to the nuclear compartment soon follows via an importin protein-independent mechanism [15–20]. However, subsequent studies have demonstrated cytoplasmic release of active recombinant protein [21].

Here, we implemented a similar strategy to dissect the early molecular mechanisms of iPSC derivation, namely, the contribution of each reprogramming factor in suppression of the murine fibroblast lineage gene Thy1 that characterizes reprogramming of transgenic (Oct4-GFP) mouse embryonic fibroblasts to iPSC [22] and exploited the ease of manipulation of protein delivery to attempt to maximize reprogramming efficiency through a staggered approach of reprogramming factor expression/exposure. Conceptually, advantages associated with utilizing protein delivery to dissect molecular mechanisms of reprogramming include its ready reversibility, allowing transient treatment of known and/or bolus quantities of protein, and the ability to circumvent the lag in transcription and translation inherent in constitutive and inducible proviral strategies. We further describe an alternative (fusion) protein purification approach, purifying and concentrating soluble (nondenatured) pTAT-mcMyc protein from induced bacterial cells, and deliver particulate/semisoluble protein of known concentrations.

## 2. Materials and Methods

### 2.1. Materials

All reagents were purchased from Sigma-Aldrich (Castle Hill, NSW, Australia) unless otherwise stated. All sequencing was performed at the Gandel Sequencing Facility, Monash Institute of Medical Research, Australia.

### 2.2. Methods

#### 2.2.1. Mice and Animal Ethics

Experiments were approved by the Monash University Animal Ethics Committee and satisfied Australian National Health and Medical Research Council (NH&MRC) guidelines for animal experimentation. MEF were harvested from 13.5 dpc OG2 × OG2 mice harboring a GFP reporter expressed from Oct4 proximal and distal enhancers and the Oct4 promoter proper [23]. All experiments were conducted using MEF passage 3.

#### 2.2.2. Construction of Tat Expression Vectors

PCR products were amplified (High Fidelity PCR protocol; Roche, Australia) from pMXs plasmid template encoding cDNA for mouse cMyc (mcMyc; Addgene, USA). Primers contributed restriction enzyme digest sites with adjacent linker DNA and stop sequences (where applicable) to PCR product (outlined by supplemental Figure 1C of the supplementary material available online at doi: 10.1155/2012/541014). Amplified PCR products and pTAT expression vector (generously provided by Dr Stephen F. Dowdy, University of California/Howard Hughes Medical Institute, USA) underwent overnight digestion (4°C) with restriction enzymes *EcoR1* and *Xho1* (Biolabs, Australia) before agarose gel purification and isolation (QIAgen Australia). Digested PCR products and pTAT plasmid were ligated with T4 DNA ligase (Promega, Australia) via manufacturer's conditions (overnight, 4°C), before electro-transformation to DH10B competent cells (BioRAD, Australia). Transformation preparations were spread to agar plates under 50 *μ*g/mL kanamycin selection and clones screened for successful ligation by sequencing (T7 sequencing primer).

#### 2.2.3. Expression, Purification, and Concentration of 6XHis-Tagged, pTAT-mcMyc Fusion Protein

Ligated pTAT-mcMyc plasmid, and native pTAT plasmid (control) were electro-transformed to BL21 (DE3) competent cells (Stratagene, USA) and spread on agar under kanamycin selection. Clones were expanded in LB Broth (made in house) containing kanamycin selection and sequenced again. Protein expression was performed by 0.1 mM isopropyl *β*-D-thiogalactosidase-(IPTG-)induced protein expression (230 rpm agitation overnight, 37°C). Initially, we purified soluble and insoluble (requiring purification of denatured protein) fractions of pTAT-mcMyc and control pTAT protein before analysis by Western Blot (purified Ni-NTA columns; QIAgen, Australia). We confirmed pTAT-mcMyc protein predominantly present in the soluble fraction by Western Blot (outlined below).

To purify soluble pTAT-mcMyc and pTAT control proteins, bacteria were lysed (1% Triton X-100, 0.1 mg/mL Lysozyme in 50 mM NaH_2_PO_4_, 300 mM NaCl, 10 mM imidazole, pH 8.0) and incubated at 37°C for 1 hour, and on ice for 30 mins. RNase (5 *μ*g/mL; Invitrogen, Australia), DNase (2 units/mL; Promega, Australia) in 1 mM MgCl_2_ and phenylmethylsulfonyl fluoride (1 mM) were added before sonication on ice. Cells were further homogenized through a 23-gauge needle before centrifugation (9000 xg, 30 mins, 4°C). The supernatant was collected for purification/elution through Ni-NTA columns via manufacturer's instructions.

To concentrate protein preparations, four-part ice-cold acetone was added to one part purified protein (v/v) and incubated at −20°C for 30–45 minutes. Following centrifugation at 6800 xg for 10 minutes (4°C), proteins pellets were resuspended in 50 *μ*L sterile H_2_O or PBS. Molar concentrations of proteins were calculated against known protein standards with the colorimetric BioRAD ^D^_C_ Protein Assay (for pTAT control protein; BioRAD, Australia), or by protein spectrophotometry (for pTAT-mcMyc).

#### 2.2.4. Western Blot

Reduced and denatured pTAT-mcMyc and control pTAT protein was electrophorated (90 V, ~2 hour, 4°C) through 12–15% denaturing polyacrilymide gel and either (a) fixed in 10% methanol /7% acetic acid before staining with SYPRO Ruby whole protein stain (BioRAD, Australia) and visualization under UV light, or (b) blotted to PVDF membrane (Millipore, Australia). Blotted wet membrane was blocked with Odyssey blocking Buffer (Odyssey, Australia) and probed with anti-6xHis-tag primary antibody (1 : 2500; Sapphire/Abcam, Australia) or anti-mcMyc (target sequence CSTSSLYLQDLSAAASEC) primary antibody (1 : 50, Sapphire/Abcam, Australia). Primary antibodies were detected with the anti-mouse Alexa fluor-680 secondary antibody (Molecular Probes; Invitrogen, Australia) for 1 h at room temperature. Test and negative control membranes (secondary antibody only) were visualized on an Odyssey InfraRed Imager (LI-COR Biosciences, Lincoln, NE, USA; intensity: 3–10, quality: medium, resolution: 169). Visualized protein bands were compared to predicted molecular weights for each protein calculated with EXpasy Software (http://web.expasy.org/compute_pi/).

#### 2.2.5. Immunocytochemistry

To confirm translocation of pTAT-mcMyc and pTAT control protein to the nuclear compartment of treated cells, we performed fluorescent immunocytochemistry according to standard methods. 2 × 10^4^ MEF were plated to coverslips before 100 nM pTAT-mcMyc or pTAT control protein was applied and incubated at 37°C for 1 hour [24]. Cells were extensively washed in PBS, fixed in 4% paraformaldehyde (prepared inhouse) and blocked in 2.5% skim milk/2.5% goat serum/PBS before labeling with 6xHis (1 : 1500; Sapphire/Abcam, Australia). For confirmation of expression of pluripotency markers, iPSCs were labeled with SSEA1 (dilution 1 : 100; Chemicon/Millipore, Australia). Bound primary antibody was detected with Alexa Fluor 488 or 555 (1 : 1500; Molecular Probes/Invitrogen, VIC, Australia). Cell nuclei were detected with 1 mg/mL Bisbenzimide Hoechst 33342. Images were taken using confocal microscope with FluorView software (version 1.5 or 4.5).

#### 2.2.6. Induction of Pluripotency in MEF OSK ± pTAT-cMyc Protein Treatment

iPSCs were derived from OG2 MEF via established protocols, using pMXs retroviral vectors [1, 22]. Briefly, 3.8 × 10^4^ MEF (12-well plates) were infected with retrovirus harboring mOct4, mSox2, mKlf4 (denoted OSK forthwith) ± mcMyc packaged in Platinum-E cell [25]. Parallel infection of MEF with a GFP-reporter transgene confirmed ≥80% infection efficiency. One-part FP media (10% (v/v) fetal bovine serum (FBS; JRH, Australia), 0.5% (v/v) penicillin/streptomycin in DMEM) replaced mcMyc retrovirus where applicable. After 24-hour incubation, virus containing media was replaced with standard ES cell culture media (designated day 0, see Figure 2(a); 15% FBS, 1% (v/v) Non-essential amino acids (Invitrogen, Australia), 1% (v/v) glutamax (Gibco, Australia), 0.1% (v/v) *β*-mercaptoethanol (Gibco, Australia), and mouse LIF (Millipore/Chemicon, Australia). ES media was changed daily for 12 days. At day 12, suitable clones of each condition were picked and expanded for future analysis.

To avoid pH-related protein denaturing events, ES media was equilibrated at 37°C/5% CO_2_ for ≥1 hour before 100 nM semisoluble pTAT-cMyc or control protein was added to applicable conditions. On days 5, 7, 9, and 12 after infection (PI), Oct4-GFP^+^ colonies were counted or cells collected for flow cytometry (outlined below). 

#### 2.2.7. Alkaline Phosphatase Expression Analysis

Alkaline phosphatase expression was confirmed in 4% paraformaldehyde fixed iPSCs colonies via standard protocols (Millipore, Australia).

#### 2.2.8. Teratoma Formation Assay: Hind Leg Injection

Approximately 1 × 10^6^ iPSCs (clone TATc1) were injected into the hind leg of 2x SCID mice. Teratomas were harvested 6–8 weeks after injection and sectioned for haemotoxylin/eosin staining (Histology Facility, MIMR) and visualization.

#### 2.2.9. Flow Cytometry

Detached cells were blocked in blocking buffer (1-2% bovine serum albumin/PBS) for 15 minutes at room temperature before primary antibody was added (4.8 *μ*g/mL Thy1-PE; eBioscience, Australia). Following 30–45 minute incubation, cells were washed 3 times in blocking buffer and resuspended in PBS (without Ca^2+^/Mg^2+^). Flow cytometry and analysis was performed on Becton Dickinson BDCanto II Flow cytometer (Becton Dickinson, Australia).

#### 2.2.10. Embryo Aggregation

Zygotes (0.5 dpc) were isolated from ampullae of mated female F_1_ mice and cultured in droplets of KSOM media (Chemicon/Millipore, Australia) until they developed to the compacted morula stage at 2.5 dpc. *Zona pellucidae* were digested from embryos with a short incubation in Acid Tyrodes solution (pH 2.5) before aggregation with 10–15 TATc1 iPSC in depressions in culture dishes formed with darning needles [26]. Embryo/cell aggregates were cultured until blastocyst stage (4.5 dpc) and assessed for contribution of GFP^+^ cells to inner cell mass of aggregated embryos.

#### 2.2.11. Mycoplasma Testing

Culture media was supplemented with 100 nM pTAT-mcMyc or pTAT-control and incubated for 24 hours at 37°C. Media was collected and tested for the presence of mycoplasma. Mycoplasma testing was performed with MycoAlert Mycoplasma Detection kit (Lonza Rockland Inc, ME USA) at MIMR Histology Core facility.

#### 2.2.12. Statistical Analysis

Where variances of OSKM and other treatment groups were sufficiently different (Tukey's test), one-way ANOVA was performed on log-transformed data. Means of duplicate wells for experimental repeats of OSK and OSK + pTAT-mcMyc (Figure 2(d)) indicated within-day variability. One-way ANOVA was performed on raw data (equal variances) or log-transformed data (unequal variances) for normalized Thy1^+^ cells of each treatment group (http://faculty.vassar.edu/lowry/t_ind_stats.html).

## 3. Results

### 3.1. Individual, and Combinations, of Reprogramming iPSC Factors Downregulate Thy1 to Varying Degrees

**Figure 1:**
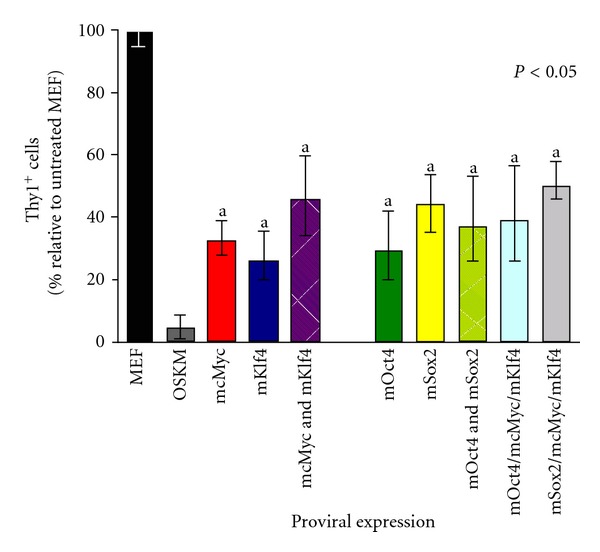
Contribution of individual reprogramming factors to repression of Thy1. MEFs were infected with either all four reprogramming factors (OSMK), individual reprogramming factors (mOct4 or mSox2 or mcMyc, or mKlf4), or combinations of reprogramming factors (mcMyc & mKlf4, OS, OMK, SMK). On day 12 after infection, the percentage of cells expressing the fibroblast marker Thy1 was assessed by flow cytometry in all treatment groups. Mean percentage Thy1^+^ cells, normalized to untreated MEF controls (black bar) of *n* = 5 experiments ± SEM are shown. Statistical significance established by one-way ANOVA, with “a” denoting groups insignificantly different from each other.

To highlight the effect of each combinations of reprogramming factor/s to Thy1 repression over 12 days, MEFs were infected with retrovirus harboring transgenes for individual or combinations of reprogramming factors before assessment for Thy1 expression by flow cytometry at day 12 (*n* = 3–5). Unsurprisingly, OSKM-expressing cells almost totally downregulated Thy1 over 12 days. All individual reprogramming factors, and combinations of ≤3 factors, suppressed Thy1 between approximately 50 and 75% of MEF over 12 days (Figure 1), although insignificantly different from each other. Although OSKM expression almost entirely extinguished Thy1 expression over 12 days, the capacity of individual Klf4 or cMyc factors to suppress Thy1 was not significantly enhanced when both factors were combined with either Oct4 or Sox2. Similarly, no additive Thy1 repression was observed when Oct4 and Sox2 factors were combined, or when cMyc and Klf4 were expressed in the same MEF (Figure 1). This suggests that maximal Thy1 repression can only be achieved in the presence of all four reprogramming factors, and not ≤3 factors (Figure 1).

### 3.2. Construction of pTAT-mcMyc Expression Vector and Subsequent Protein Expression

**Figure 2:**
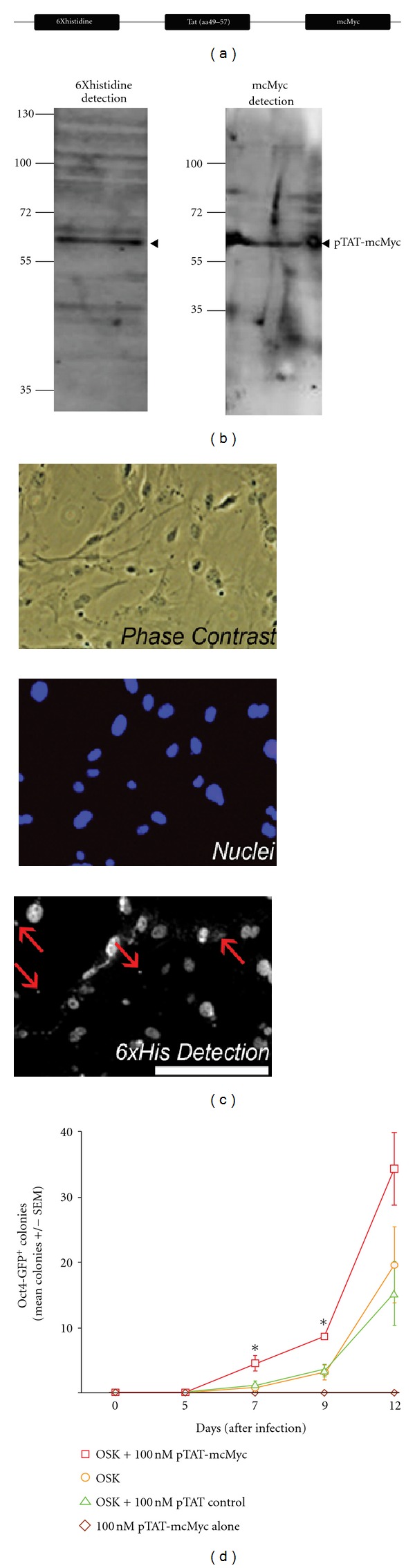
Construction and transduction of of pTAT-mcMyc recombinant protein to MEF *in vitro*. (a) Schematic representation of the pTAT-mcMyc construct. (b) Reduced and denatured pTAT-mcMyc recombinant protein was resolved on 12–15% SDS-polyacrylamide gel and transferred to PVDF membrane by electrophoresis (90 V, 2 hours, 4°C). Wet membrane was blocked for 1 hour in Odyssey blocking buffer (1 : 1 Tris-HCl buffer) and probed with either mouse anti-6xHistidine or mouse anti-mouse cMyc primary antibody before detection with goat anti-mouse Alexa Fluor-680 secondary antibody. Recombinant pTAT-mcMyc was detected at predicted molecular weight (57–60 kDa; arrow). (c) MEFs were plated to coverslips before 100 nM pTAT-mcMyc recombinant protein was applied to equilibrated culture media. Following a 1-hour incubation, MEFs were washed, fixed, blocked, and probed with antibody recognizing 6xHistidine (bottom panel). Cell nuclei were detected with Hoechst dye (middle panel). Scale bar: 200 *μ*M. Red arrows shown detection of pTAT-mcMyc protein outside of detectable nuclei. (d) MEFs were infected with retrovirus harboring OSK or left uninfected. OSK-expressing MEFs were treated with 100 nM pTAT-mcMyc protein (red square) or pTAT-control protein (green triangle) daily for 12 days. Uninfected MEFs were left as a control, or treated with 100 nM pTAT-mcMyc protein (brown diamond) daily for 12 days. On days 5, 7, 9, and 12 after infection, Oct4-GFP^+^ colonies were counted in duplicate wells. Mean ± SEM of *n* = 4 independent experiments shown; *P* < 0.05.

Control over expression levels, as well as timing of expression, is limited using retroviral strategies. Therefore we adopted a recombinant protein strategy to dissect the molecular and temporal mechanisms of reprogramming. We amplified cDNA for mouse cMyc (mcMyc) flanked by an N-terminal EcoRI restriction enzyme digest site and a C-terminal XhoI site (linked by a single guanine to maintain in-frame mcMyc sequence; primers outlined in supplemental Figure 1(C)). Following direct restriction digestion of PCR products and subsequent DNA ligation, sequencing confirmed successful, in-frame ligation of mcMyc cDNA insert to digested pET28b TAT plasmid; denoted pTAT-mcMyc forthwith (Figure 2(a)). pTAT-mcMyc and empty vector (control) plasmid were transformed to BL21(DE3) cells for IPTG-induced protein expression. We initially purified pTAT-mcMyc protein under either soluble or insoluble (denaturing) conditions and employed SDS-PAGE to confirm which fraction the recombinant proteins (of predicted molecular weight) was present in (predicted pTAT-mcMyc 53.05 kDa; EXpasy software; http://web.expasy.org/compute_pi/). Protein concentration was equated by colorimetric protein assay or spectrophotometry before freezing aliquoted protein. Reduced and denatured pTAT-mcMyc protein (of both purifying preparations) was electroporated through 12–15% polyacrilymide gel and blots probed with antibodies recognizing either (i) the 6xHistidine leader sequence (Figure 2(b) and supplemental Figure 1(A)), or (ii) amino acids 186–203 of mouse/human mcMyc (Figure 2(b)). Contrary to previous reports, we detected a primary band of purified, histidine-tagged pTAT-mcMyc protein close to predicted molecular weight primarily in the soluble fraction using antibody detecting the leader sequence (Figure 2(b)), with little detectable protein in denaturing conditions (supplemental Figure 1(A)). To confirm specificity of our anti-Histidine antibody, western blots were repeated using antibody directed against mouse/human cMyc; again, protein was detected at the same MW (Figure 2(b)). Expression of control protein of predicted molecular weight (6.099 kDa) was confirmed by His_6_ leader sequence detection (data not shown). These results suggest successful construction, expression, and purification of semisolubilized, particulate pTAT-mcMyc and pTAT-control protein close to predicted molecular weight in our bacterial expression system.

Initial experiments confirmed the pH of culture media reduced in alkalinity to near neutral after 1-hour incubation at 37°C/5% CO_2_. Therefore, to avoid denaturation of recombinant protein upon application to acidic culture media, neutrality was established prior to application of pTAT-mcMyc application by 1 hour incubation at 37°C/5% CO_2_. To ascertain whether eluted pTAT-mcMyc protein (and pTAT control protein) can transduce to the nucleus of MEF, 100 nM of pTAT-mcMyc (or 100 nM control protein) was added to cultures of MEF grown on coverslips and incubated for 1 hour. A concentration of 100 nM was adopted from a survey of previous reports for TAT fusion protein delivery [27–30]. Fixed and permeabilized cells were labeled with antibody recognizing the 6xHistidine sequence. pTAT-mcMyc and pTAT-control proteins were detected primarily confined to the nuclear compartment of treated MEF, (compare “Nuclei” and “6xHis Detection” panels; Figure 2(c)). Minimal protein was observed in cytoplasm/cytoplasmic vesicles, perhaps reflective of recombinant TAT protein within cytoplasmic endosomes or in transit to the nucleus (red arrows, Figure 2(c); [15]). Detection of antibody binding in nontreated cells was minimal, suggesting minimal nonspecific binding of antibody to alternative histidine-rich proteins (data not shown). These results confirm rapid (≤1 hour) and efficient transduction of pTAT-mcMyc (and control pTAT protein) to nuclear compartment of MEF.

### 3.3. Addition of pTAT-mcMyc Protein to OSK Provirus-Expressing Cells Accelerates Oct4-GFP^+^ Colony Formation over 12 Days (after Infection)

A delayed and reduced efficiency of iPSC colony formation is observed when cMyc is omitted from the reprogramming factor cocktail [9]. We hypothesized that the addition of the pTAT-mcMyc recombinant protein to OSK-expressing MEF would result in a significant increase in Oct4-GFP^+^ colony formation. In our hands, Oct4-GFP^+^ iPSC colony formation in OSKM provirus expressing MEF was comparable to OSK-expressing cells up to 12 days, only surpassing OSK colony formation after 12 days (data not shown). Therefore, we infected Oct4-GFP transgenic MEF with retrovirus harboring transgenes for either OSK or mcMyc alone and examined OSK ± pTAT-mcMyc over 12 days. In four separate experiments, we added either 100 nM pTAT-mcMyc or pTAT control protein to duplicate wells of OSK-expressing MEF daily for 12 days and compared Oct4-GFP^+^ colony counts to OSK-expressing MEF at days 5, 7, 9, and 12 after infection (*n* = 4; Figure 2(d)). The addition of pTAT control protein to OSK-infected cells did not elicit significant improvements in Oct4-GFP^+^ colony formation over OSK-infected cells at any time point, suggesting the Tat and linker protein sequences had little effect on Oct4-GFP^+^ colony formation (see green line, Figure 2(d)). MEF treated exclusively with 100 nM pTAT-mcMyc protein alone (Figure 2(d)) and mcMyc transgene-expressing MEF (data not shown) failed to generate colonies at any time points. Statistically significant improvements in Oct4-GFP^+^ colony formation were observed at day 7 and 9 after infection (*P* < 0.05 and *P* < 0.005, resp.) in OSK-expressing cells treated with 100 nM pTAT-mcMyc protein. This suggests that nuclear-localized pTAT-mcMyc protein is biologically functional and can accelerate iPSC colony formation in OSK MEF.

### 3.4. Confirmation of Pluripotency of OSK^+^ pTAT-mcMyc-Treated Cells

**Figure 3:**
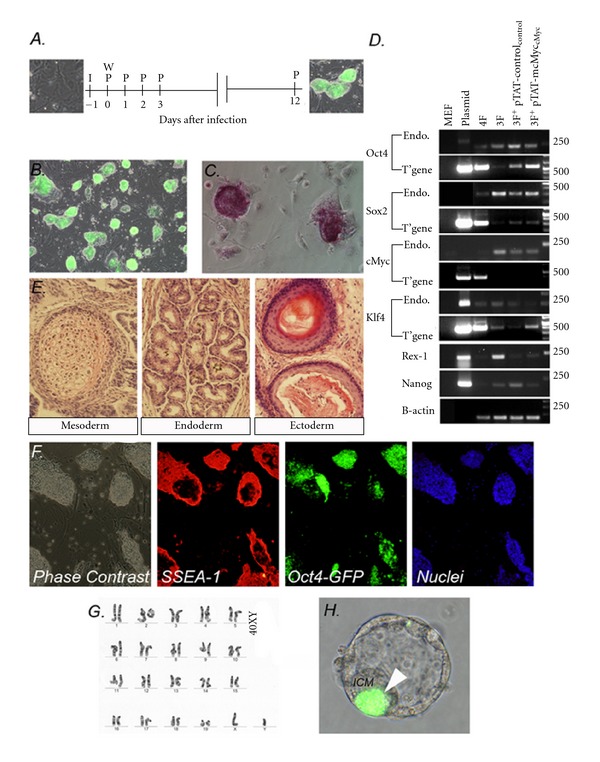
OSK + pTAT-mcMyc treated MEF display the hallmarks of pluripotency. (A) Schedule for pTAT-mcMyc treatment of somatic cells. MEF were infected for 24 hours (“I”) before retrovirus was washed from cells (“W”) and pTAT-mcMyc and control recombinant protein applied (day 0; “P”). Protein was applied daily for 12 days. (B) Oct4-GFP^+^ clone Tatc1 from Oct4/Sox2/Klf4 + pTAT-mcMycs treated cells. (C) Confirmation of alkaline phosphatase expression in clone Tatc1. (D) RT-PCR confirms Tatc1 expresses endogenous markers Oct4, Sox2, cMyc, Klf4, Rex-1, and Nanog (“Endo”). We further confirmed this clone was not contaminated with mcMyc transgene by PCR using genomic DNA template (“T'gene”; all oligonucleotides outlined elsewhere) [1]. (E) Following injection into SCID mice, Tatc1 form teratomas with differentiated cells characteristic of the three germ layers (cartilage characteristic of endoderm, glandular tissue reminiscent of mesoderm, and neural rosettes characteristic of ectoderm). (F) Immunocytochemistry confirms Tatc1 expresses pluripotency marker stage-specific embryonic antigen-1 (SSEA1). (G) Reprogramming of MEF to Tatc1 maintained normal karyotype (40XY). (H) Following aggregation with 2.5 dpc embryos, Oct-GFP^+^ Tatc1 cells contribute to the inner cell mass (labeled “ICM”) of subsequent 4.5 dpc blastocysts (arrow).

We expanded 3 × Oct4-GFP^+^ clones from experiment outlined in Figure 2(d), of which one cell line is chosen on proliferative and morphological characteristics for further analysis (denoted TATc1 forthwith; Figure 3(B). We assessed the pluripotency of the TATc1 cell line by standard pluripotency criteria. We confirmed that colonies express alkaline phosphatase (Figure 3(C)). We confirmed this clone was not contaminated with mcMyc transgene by PCR using genomic DNA template (“T”gene” in Figure 3(D)) and confirmed expression of endogenous Oct4, Sox2, Klf4, cMyc, Rex1, and Nanog (“Endo” in Figure 3(D)). Histological inspection of teratomas formed from intramuscular injection of TATc1 into the hind leg of SCID mice highlighted regions of differentiation characteristic of all three cell lineages, cartilage characteristic of mesoderm, glandular tissue reminiscent of endoderm, and neural rosettes characteristic of ectoderm (Figure 3(E)). Immunocytochemistry confirmed that compact TATc1 (Oct4-GFP^+^) colonies coexpress stage-specific embryonic antigen-1 SSEA1 (Figure 3(F)). TATc1 cells retained normal karyotype during the reprogramming period (passage 5, 20/20 counts 40XY; Figure 3(G)) and contributed to the inner cell mass of developing blastocysts when aggregated with diploid F_1_ 4–8 cell embryos (Figure 3(H)). Collectively, these results confirm that OSK + pTAT-mcMyc-treated cells share the hallmarks of pluripotency observed in OSKM- and OSK-induced cells [1, 9].

### 3.5. Addition of pTAT-mcMyc to OSK Provirus-Expressing Cells Augments Downregulation of Thy1 over 12 Days after Infection

**Figure 4:**
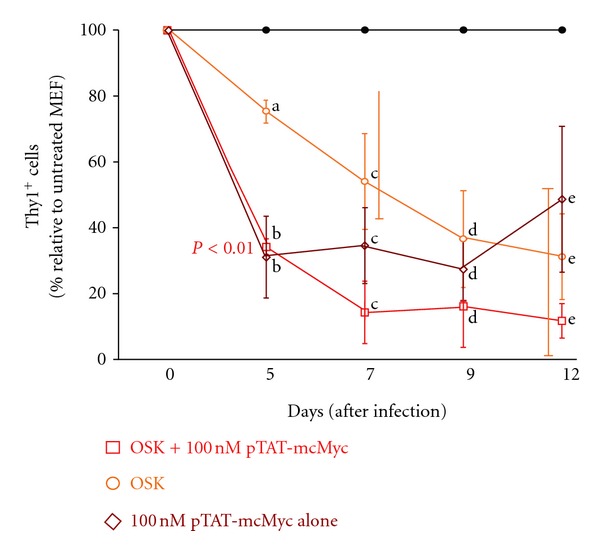
Thy1 downregulation by OSK, pTAT-mcMyc alone or OSK + pTAT-mcMyc at 5, 7, 9, and 12 days after infection. MEFs were infected with retrovirus harboring transgenes for mOct4, mSox2, and mKlf4 (OSK; orange line). 100 nM pTAT-mcMyc recombinant protein was applied to OSK expressing cells (red line), or to non-transduced MEFs (brown line). On days 5, 7, 9, and 12 after infection, percentage of cells expressing the fibroblast marker Thy1 was assessed in all treatment groups by flow cytometry with results normalized to untreated MEF controls (black line). Mean ± SEM of *n* = 2 experiments shown.

All individual reprogramming factors can repress lineage gene expression (i.e., Thy1) over a 12 days period, with ≥5 days of initial cMyc expression required for efficient AP+ colony formation (Figure 1) [8]. Hence, we adopted a recombinant protein delivery approach to dissect the temporal and molecular mechanisms of somatic cell reprogramming, primarily repression of Thy1 [12, 31]. In an independent experiment, we collected experimental groups on days 5, 7, 9, and 12 (*n* = 3) to assess the percentage of cells expressing the Thy1 by flow cytometry, normalizing results to untreated MEF controls (39.2 ± 4.7%, mean ± SEM in our hands; Figure 4). A gradual downregulation of Thy1 was observed in OSK expressing cells over 12 days post infection (Figure 4). Application of 100 nM pTAT-mcMyc protein alone to MEF elicited a significant downregulation of Thy1 over the initial 5-day treatment period, which was maintained, from 5 to 12 days (Figure 4; *P* < 0.05). Improved (although not significant) Thy1 repression continued to 7 days in MEF expressing OSK and treated with 100 nM pTAT-mcMyc protein, with Thy1 repression maintained to 12 days after infection (Figure 4). Mycoplasma testing of protein-treated culture media confirmed Thy1 repression was not due to contaminating mycoplasma from protein purification (data not shown).

### 3.6. Five-Day Pretreatment of OG2 MEF with pTAT-mcMyc ± Exogenous mKlf4 Expression for Subsequent mOct4/mSox2-Mediated Reprogramming

**Figure 5:**
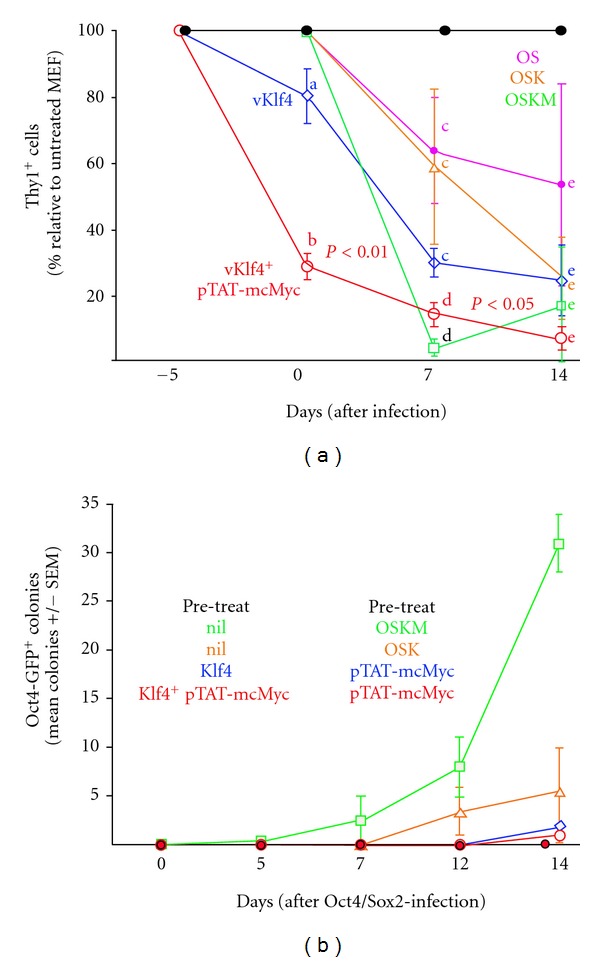
A staggered approach to application of reprogramming factors. (a) MEF were pre-treated with mKlf4 (provirus) ± pTAT-mcMyc protein (or untreated) for 5 days and infected with mOct4 and mSox2 at day 0. Alternatively, MEF were not pre-treated and infected with mOct4+mSox2, mOct4+mSox2+mKlf4 or all four reprogramming factors (OSKM). The percentage of Thy1^+^ MEF was ascertained relative to untreated MEF at days 0, day 7 and day 14 post-infection. Statistical significance in percentage of Thy1^+^ MEF was assessed by one-way ANOVA. Mean ± SEM of *n* = 3 experiments shown. (b) Oct4-GFP^+^ colony counts for experiments outlined in 6A were counted at days 0, 5, 7, 12, and 14 post-infection. Mean ± SEM of *n* = 3 experiments shown.

Lineage gene repression early in reprogramming is a prerequisite for pluripotency gene activation late in reprogramming [4, 5]. The dispensability of cMyc from the initial 5 days of reprogramming suggests a role in lineage gene repression for this reprogramming factor [8]. Since significant Thy1 repression results from pTAT-mcMyc recombinant protein application (in presence or absence of Klf4 expression; Figure 4), we proposed a staggered approach to initiating reprogramming; a 5-day pretreatment (and therefore “prerepression” of Thy1) of MEF with Klf4 ± 100 nM pTAT-mcMyc recombinant protein could facilitate subsequent accelerated Oct4 + Sox2-mediated reprogramming (assessed by Thy1 downregulation, Figure 5(a); Oct4-GFP colony formation, Figure 5(b)). Therefore, we infected OG2 MEF with retrovirus harboring Klf4 transgene before incubation in the presence or absence of 100 nM pTAT-mcMyc for 5 pretreatment days (designated: 5 days; Figure 5(a)). Alternatively, MEF remained uninfected and untreated for the pretreatment period. After 5 days of Klf4 expression ± recombinant protein treatment (designated day 0), treatment groups and untreated MEFs were (i) collected and analysed by FACS for proportion of Thy1^+^ cells and (ii) replated for a second infection of retrovirus for mOct4 and mSox2. As controls, nontreated MEF were plated for infection with retrovirus for (i) mOct4 and Sox2 only, (ii) mOct4, mSox2 and mKlf4 only, or (iii) mOct4, mSox2, mKlf4, and mcMyc (Figure 5). FACS analysis for Thy1^+^ cells was performed on days 0, 7, and 14 after mOct4/mSox2 infection with percentage Thy1^+^ cells in each treatment group normalized to untreated control MEF. Oct4-GFP colony counts were also counted at same time points to assess effect of pre/posttreatment on reporter gene^+^ colony formation (Figure 5(b)).

As highlighted in Figure 5, five days pretreatment/expression of (i) exogenous Klf4 and (ii) exogenous Klf4 + 100 nM pTAT-mcMyc resulted in repression of Thy1 in 20–70% of MEF, respectively (designated day 0, Figure 5(a)). Application of 100 nM pTAT-mcMyc significantly enhanced Thy1 repression in Klf4-expressing MEF (*P* < 0.01; Figure 5(a)). Untreated MEF were infected with OS, OSK, or OSKM (pink, orange, and green lines, resp.) and pretreated MEF were infected with OS only, with pTAT-mcMyc pretreatment continuing in pretreated MEF (red line).

After 7-day expression, Thy1 repression in OS- and OSK-infected (only) MEF was modest (in 35–40% of MEF). Thy1 expression was almost completely abolished in MEF infected with OSKM (i.e., not prereated) at day 0. Continued 100 nM pTAT-mcMyc treatment in Klf4 preinfected/Oct4/Sox2 postinfected MEF elicited continued Thy1 repression to day 7 to a level insignificantly different from MEF expressing all four reprogramming factors. Interestingly, preinfection with Klf4 (thus prerepressing Thy1), with subsequent Oct4/Sox2 infection, was equally effective at repressing Thy1 at 14 days than infecting cells concurrently with OSK (compare blue and orange lines; Figure 5(a)).

Concurrent OSKM (significant) and concurrent OSK (insignificant) infection still yielded more colonies than the staggered approach adopted above (Figure 5(b)). In fact, few Oct4-GFP^+^ colonies were observed for any of the Klf4 ± 100 nM pTAT-mcMyc pretreated/Oct4 + Sox2 postinfected groups. These results suggest that although increased Thy1 repression is achieved through pretreatment with Klf4 ± 100 nM pTAT-mcMyc, concurrent infection with all four reprogramming factors still yields most efficient reporter gene^+^ colonies at day 14. At 14 days, pretreatment of MEF with exogenous Klf4 + 100 nM pTAT-mcMyc before Oct4/Sox2 infection at day 0, significantly repressed Thy1 than cells infected with Oct4/Sox2 alone. However, this effect is not reflected in GFP^+^ colony counts at day 14.

## 4. Discussion

To highlight the contribution of each/combinations of reprogramming factors, and possible suppressive effects of each capability to repress Thy1, we infected MEF with individual reprogramming factors or combinations of factors and assessed Thy1 at day 12 after infection (Figure 1). Twelve-day expression of each individual reprogramming factors effectively downregulates Thy1 (Figure 1). We failed to observe an additive effect of expressing both Oct4 and Sox2 compared with expressing either factor alone, or expressing both cMyc and Klf4 rather than expressing either factor alone. The capacity of Oct4 or Sox2 to individually downregulate Thy1 was not significantly enhanced when (i) expressed concurrently, or when (ii) cMyc and Klf4 were also expressed in the same MEF (Figure 1).

We adopted a recombinant protein delivery approach to dissect the molecular mechanisms of somatic cell reprogramming. We describe a method for generating semisoluble, particulate recombinant mcMyc protein with an N-terminal linked 11-amino-acid, arginine-rich motif (^49^ **R**KK**RR**Q**RRR** ^57^) of transactivating transcriptional-activator (Tat) of HIV (Figure 2(a)) [21, 32]. The TAT and mcMyc functional domains were coupled through a peptide linker sequence, reducing interference between these domains [14]. Detection of pTAT-mcMyc protein with antibodies recognizing either the 6xHistidine leader sequence or cMyc protein by western blot analysis confirmed expression and purification of in-frame recombinant protein of predicted molecular weight (Figure 2(b)). Initial experiments suggested pTAT-mcMyc protein was primarily soluble, and particulate when resuspended after acetone precipitation (data not shown). Binding of the Tat domain to heparan sulfate proteoglycan of cells *in vitro* initiates rapid (in the order of minutes) translocation of recombinant protein to the nuclear compartment (Figure 2(c)) [15–20, 33].

Notable differences in protein purification and concentration distinguish published reports from the present study, with recombinant proteins commonly purified from bacterial inclusion bodies before refolding [13, 14]. Kim et al. [12], expressed proteins in transfected human cells and applied unknown quantities of unpurified reprogramming factors in whole protein extracts to targets cells, with neither study concentrating recombinant protein fractions/preparations. Purification of soluble, un-denatured protein in the present study circumvents potential problems associated with misfolding of recombinant protein to numerous alternative (and potentially inactive) conformations, with acetone concentration removing nonprotein bacterial contaminants [14, 34, 35]. Previous attempts to utilize denatured Tat-fusion protein for reprogramming of human fibroblasts were hampered by restriction of protein to endocytotic vesicles [14]. Treatment of target cells in serum-free conditions may also restricted cytoplasmic vesicular release. Purification of semisoluble, particulate pTAT-mcMyc recombinant protein, and/or equilibration of culture media before recombinant protein application, may contribute to escape and/or evasion of our Tat-fusion proteins from such endocytotic vesicles [14].

We applied 100 nM pTAT-mcMyc protein to uninfected MEF or OSK-expressing MEF daily for 12 days and monitored Oct4-GFP^+^ colony formation (Figure 2(d)) [32, 36, 37]. Contrary to previous reports demonstrating toxicity of 80 nM TAT-DsRED-Klf4 application in MEF [14], we failed to observe adverse effects in MEF cultured in 100 nM pTAT-mcMyc. This may be attributable to cytotoxicity observed in a number of red fluorescent protein (RFP) variants, or unexplained toxicity of Klf4 protein itself at these concentrations. Nonetheless, up to day 5 after infection, ES-like Oct4-GFP^+^colonies were not observed in any treatment group (Figure 2(d)) [9]. Significantly accelerated Oct4-GFP^+^ colony formation was observed in OSK-expressing cells treated with pTAT-mcMyc at days 7 and 9 after infection. Clone Tatc1 expressed (Oct4)-GFP and alkaline phosphatase, differentiated to all germ layers in teratomas, expressed pluripotency markers (as assessed by RT-PCR and immunocytochemistry), maintained normal karyotype and was capable of contributing to the inner cell mass of aggregated chimeric embryos (Figures 3(A)–3(H)). This confirms conversion of OSK + pTAT-mcMyc recombinant protein-treated MEF to a fully reprogramming phenotype.

We combined transgene expression ± pTAT-mcMyc recombinant protein delivery to highlight mechanisms of lineage gene repression. Thy1 (CD90) is glycosylphosphatidylinositol-anchored plasma membrane glycoprotein expressed in a variety of cell types (including fibroblast populations) implicated in cell proliferation and apoptosis, cytoskeletal organization, cell-cell/matrix adhesion, and a number of cytoplasmic signaling cascades [6, 7, 38]. Since constitutive expression of OSKM transgenes represses Thy1 in the majority of murine fibroblasts, we exploit the ability to apply controlled concentrations of proteins over defined periods to highlight mechanisms of Thy1 repression [5]. Five days of 100 nM pTAT-mcMyc recombinant protein treatment (±OSK expression) initiates considerable repression of Thy1 expression (Figure 4; as assessed by flow cytometry). OSK transgene expression also elicits downregulation of Thy1, but at markedly reduced efficiency. Surprisingly, combining OSK expression and pTAT-mcMyc delivery failed to elicit additive Thy1 repression on OSK alone. This result suggests that mcMyc primarily mediates Thy1 repression <5 days in the presence or absence of OSK, overriding or “saturating” the moderate OSK-mediated repression or is utilized preferentially when “expressed” in the same cell, thus suggesting Thy1 repression in the initial stages of reprogramming may be rate limiting. Perhaps OSK-mediated Thy1 repression is the default mechanism for early reprogramming, when sufficient concentrations of mcMyc are absent. It is unclear, yet possible, that cMyc directly or indirectly promotes recruitment of histone methyltranferase/s to the Thy1 promoter to initiate transcriptional repression, or disrupt cytoskeletal actin bundles to permit cellular morphological changes and adoption of pluripotent phenotype [38–40]. The transition from Thy1^+^ to a Thy1^−^ phenotype in nasopharyngeal mucosa is a feature of carcinogenesis, and thus suggests that Thy1 represents a candidate as a tumor suppressor [41]. Given the similarities in genetic profile between embryonic stem cells and cancer stem cells, it is unsurprising that repression of tumour-repressor gene function/immortalization markedly increases the efficiency of somatic cell reprogramming [42–44]. Lung fibroblast populations lacking Thy1 have considerably reduced methyltransferase levels ascertained by real-time PCR, with chemically induced demethylation of Thy1^−^ fibroblasts reinitiating Thy1 expression [39].

A gradual trend (although statistically insignificant) of Thy1 downregulation is observed in OSK expressing cells between 5 and 12 days (Figure 4). By contrast, pTAT-mcMyc protein alone fails to mediate significant improvements in Thy1 downregulation over the same period and is not a significant improvement on OSK-mediated Thy1 suppression. However, addition of 100 nM pTAT-mcMyc protein to OSK provirus-expressing MEF further promotes Thy1 suppression to 7 days after infection, an improvement approaching statistical significance on OSK alone (*P* = 0.07; Figure 4). A cooperative or additive mechanism between 100 nM pTAT-mcMyc and either O ± S ± K may accelerate Thy1 repression from 5 to 7 days.

The reversibility of recombinant protein delivery, as well as ability to control the temporal and concentration aspects of its application, permits the manipulation of the iPS reprogramming methodology with a view to improving reprogramming efficiency. cMyc is required for the initial days of reprogramming only (for AP^+^ colonies), yet Oct4 is required throughout reprogramming [8]. Since lineage gene repression is an early prerequisite for reprogramming, we hypothesized a staggered approach, that is, prerepressing Thy1 through application of Klf4 ± pTAT-mcMyc with subsequent proviral expression of Oct4 and Sox2, could improve reprogramming efficiency, as assessed by Oct4-GFP^+^ colony formation. This may also reduce the time required to expose target cells with reprograming factors, in particular Oct4 and Sox2. Although 5-day application of Klf4 ± pTAT-mcMyc significantly repressed Thy1 at the time of Oct4/Sox2 application (day 0), total Thy1 repression was comparable to consecutive 4-factor expression at 14 days (Figure 5(a)). Importantly, pre-repression of Thy1 did not improve Oct4-GFP^+^ colony formation (Figure 5(b)). Although Thy1 expression is repressed with equal effectiveness over 7 and 14 days by (i) consecutive OSKM expression or (ii) staggered expression Klf4/pTAT-mcMyc and Oct4/Sox2 (Figure 5(a)), it does not translate to Oct4-GFP^+^ colony formation at any time point (Figure 5(b)). In fact, Oct4-GFP^+^ colony formation was also delayed in pretreated MEF (Figure 5(b)). These results suggest although Thy1 repression is a pre-requiste for subsequent pluripotency gene activation and successful reprogramming, with cMyc and Klf4 primarily responsible for mediating this early event (Figures 4 and 5) [4, 5, 8], early coexpression of Oct4 and Sox2 is still required for timely and efficient reporter gene^+^ colony formation later in reprogramming (i.e., 14 days).

To conclude, we have exploited the endocytotic properties of Tat transduction peptide to rapidly transport key transcription factors to the nuclei of Oct4-GFP MEF. Experimental results outlined here suggest mcMyc regulates the initial (<5 days) suppression of Thy1 in MEF, followed by a cooperative mechanism driven by mcMyc/Klf4 from 5–9 days after infection (Figure 4). Protein delivery can be transduced to mitotically active or inactive cells, or cells that are difficult to infect with cDNAs (e.g., mesenchymal stem cells), does not permanently disrupt the host genome and can be transiently applied to drive reprogramming and is rapidly reversible. Therefore, we suggest application of additional Tat fusion constructs at varying concentrations and times will further highlight the events that characterize somatic cell reprogramming. As heparan sulfate proteoglycan display considerable sequence homology between mammalian species and are ubiquitously expressed throughout many cell types, the same Tat fusion constructs could potentially be used in many cells types within and between mammalian species and be used to generate protein-iPS cells in a controlled manner. We anticipate more studies dedicated to varying concentrations of recombinant proteins to highlight threshold levels of reprogramming factors to maximize somatic cell reprogramming.

## References

[1] Takahashi K, Yamanaka S (2006). Induction of pluripotent stem cells from mouse embryonic and adult fibroblast cultures by defined factors. *Cell*.

[2] Okita K, Nakagawa M, Hyenjong H, Ichisaka T, Yamanaka S (2008). Generation of mouse induced pluripotent stem cells without viral vectors. *Science*.

[3] Heffernan C, Sumer H, Verma PJ, Rosales DW, Mullen QN (2009). Generation of clinically relevant induced pluripotent stem (iPS) cells. *Pluripotent Stem Cells*.

[4] Brambrink T, Foreman R, Welstead GG (2008). Sequential expression of pluripotency markers during direct reprogramming of mouse somatic cells. *Cell Stem Cell*.

[5] Stadtfeld M, Maherali N, Breault DT, Hochedlinger K (2008). Defining molecular cornerstones during fibroblast to iPS cell reprogramming in mouse. *Cell Stem Cell*.

[6] Koumas L, Smith TJ, Feldon S, Blumberg N, Phipps RP (2003). Thy-1 expression in human fibroblast subsets defines myofibroblastic or lipofibroblastic phenotypes. *The American Journal of Pathology*.

[7] Rege TA, Hagood JS (2006). Thy-1, a versatile modulator of signaling affecting cellular adhesion, proliferation, survival, and cytokine/growth factor responses. *Biochimica et Biophysica Acta*.

[8] Sridharan R, Tchieu J, Mason MJ (2009). Role of the murine reprogramming factors in the induction of pluripotency. *Cell*.

[9] Nakagawa M, Koyanagi M, Tanabe K (2008). Generation of induced pluripotent stem cells without Myc from mouse and human fibroblasts. *Nature Biotechnology*.

[10] Judson RL, Babiarz JE, Venere M, Blelloch R (2009). Embryonic stem cell-specific microRNAs promote induced pluripotency. *Nature Biotechnology*.

[11] Smith KN, Singh AM, Dalton S (2010). Myc represses primitive endoderm differentiation in pluripotent stem cells. *Cell Stem Cell*.

[12] Kim D, Kim CH, Moon JI (2009). Generation of human induced pluripotent stem cells by direct delivery of reprogramming proteins. *Cell Stem Cell*.

[13] Zhou H, Wu S, Joo JY (2009). Generation of induced pluripotent stem cells using recombinant proteins. *Cell Stem Cell*.

[14] Pan C, Lu B, Chen H, Bishop CE (2010). Reprogramming human fibroblasts using HIV-1 TAT recombinant proteins OCT4, SOX2, KLF4 and c-MYC. *Molecular Biology Reports*.

[15] Frankel AD, Pabo CO (1988). Cellular uptake of the tat protein from human immunodeficiency virus. *Cell*.

[16] Green M, Loewenstein PM (1988). Autonomous functional domains of chemically synthesized human immunodeficiency virus tat trans-activator protein. *Cell*.

[17] Efthymiadis A, Briggs LJ, Jans DA (1998). The HIV-1 tat nuclear localization sequence confers novel nuclear import properties. *Journal of Biological Chemistry*.

[18] Rusnati M, Tulipano G, Spillmann D (1999). Multiple interactions of HIV-I Tat protein with size-defined heparin oligosaccharides. *Journal of Biological Chemistry*.

[19] Tyagi M, Rusnati M, Presta M, Giacca M (2001). Internalization of HIV-1 Tat requires cell surface heparan sulfate proteoglycans. *Journal of Biological Chemistry*.

[20] Fittipaldi A, Ferrari A, Zoppé M (2003). Cell membrane lipid rafts mediate caveolar endocytosis of HIV-1 Tat fusion proteins. *Journal of Biological Chemistry*.

[21] Tang Y, Lin C-J, Tian XC (2011). Functionality and transduction condition evaluation of recombinant klf4 for improved reprogramming of iPS cells. *Cellular Reprogramming*.

[22] Tat PA, Sumer H, Jones KL, Upton K, Verma PJ (2010). The efficient generation of induced pluripotent stem (iPS) cells from adult mouse adipose tissue-derived and neural stem cells. *Cell Transplantation*.

[23] Szabó PE, Hübner K, Schöler H, Mann JR (2002). Allele-specific expression of imprinted genes in mouse migratory primordial germ cells. *Mechanisms of Development*.

[24] Jiang L, Ma Y, Wang J, Tao X, Wei D (2008). The transduction of His-TAT-p53 fusion protein into the human osteogenic sarcoma cell line (Saos-2) and its influence on cell cycle arrest and apoptosis. *Molecular Biology Reports*.

[25] Morita S, Kojima T, Kitamura T (2000). Plat-E: an efficient and stable system for transient packaging of retroviruses. *Gene Therapy*.

[26] Eakin GS, Hadjantonakis AK (2006). Production of chimeras by aggregation of embryonic stem cells with diploid or tetraploid mouse embryos. *Nature Protocols*.

[27] Mayne M, Holden CP, Nath A, Geiger JD (2000). Release of calcium from inositol 1,4,5-trisphosphate receptor-regulated stores by HIV-1 Tat regulates TNF-*α* production in human macrophages. *Journal of Immunology*.

[28] Self RL, Mulholland PJ, Nath A, Harris BR, Prendergast MA (2004). The human immunodeficiency virus type-1 transcription factor Tat produces elevations in intracellular Ca^2+^ that require function of an N-methyl-D-aspartate receptor polyamine-sensitive site. *Brain Research*.

[29] Zhong Y, Smart EJ, Weksler B, Couraud PO, Hennig B, Toborek M (2008). Caveolin-1 regulates human immunodeficiency virus-1 Tat-induced alterations of tight junction protein expression via modulation of the ras signaling. *Journal of Neuroscience*.

[30] Thier M, Münst B, Edenhofer F (2010). Exploring refined conditions for reprogramming cells by recombinant Oct4 protein. *International Journal of Developmental Biology*.

[31] Herce HD, Garcia AE, Litt J (2009). Arginine-rich peptides destabilize the plasma membrane, consistent with a pore formation translocation mechanism of cell-penetrating peptides. *Biophysical Journal*.

[32] Nagahara H, Vocero-Akbani AM, Snyder EL (1998). Transduction of full-length TAT fusion proteins into mammalian cells: TAT-p27(Kip1) induces cell migration. *Nature Medicine*.

[33] Yun CO, Shin HC, Kim TD (2008). Transduction of artificial transcriptional regulatory proteins into human cells. *Nucleic Acids Research*.

[34] Dobson CM (2003). Protein folding and misfolding. *Nature*.

[35] Jiang L, He L, Fountoulakis M (2004). Comparison of protein precipitation methods for sample preparation prior to proteomic analysis. *Journal of Chromatography A*.

[36] Dolgilevich S, Zaidi N, Song J, Abe E, Moonga BS, Sun L (2002). Transduction of TAT fusion proteins into osteoclasts and osteoblasts. *Biochemical and Biophysical Research Communications*.

[37] Clohisy JC, Hirayama T, Frazier E, Han SK, Abu-Amer Y (2004). NF-kB signaling blockade abolishes implant particle-induced osteoclastogenesis. *Journal of Orthopaedic Research*.

[38] Barker TH, Grenett HE, MacEwen MW (2004). Thy-1 regulates fibroblast focal adhesions, cytoskeletal organization and migration through modulation of p190 RhoGAP and Rho GTPase activity. *Experimental Cell Research*.

[39] Sanders YY, Pardo A, Selman M (2008). Thy-1 promoter hypermethylation: a novel epigenetic pathogenic mechanism in pulmonary fibrosis. *American Journal of Respiratory Cell and Molecular Biology*.

[40] Martinato F, Cesaroni M, Amati B, Guccione E (2008). Analysis of myc-induced histone modifications on target chromatin. *PLoS ONE*.

[41] Hong LL, Bangarusamy DK, Xie D (2005). THY1 is a candidate tumour suppressor gene with decreased expression in metastatic nasopharyngeal carcinoma. *Oncogene*.

[42] Wong DJ, Liu H, Ridky TW, Cassarino D, Segal E, Chang HY (2008). Module map of stem cell genes guides creation of epithelial cancer stem cells. *Cell Stem Cell*.

[43] Utikal J, Polo JM, Stadtfeld M (2009). Immortalization eliminates a roadblock during cellular reprogramming into iPS cells. *Nature*.

[44] Zhao RC, Zhu YS, Shi Y (2008). New hope for cancer treatment: exploring the distinction between normal adult stem cells and cancer stem cells. *Pharmacology and Therapeutics*.

